# P-964. Case-Based Learning Outcomes for Pre-Exposure Prophylaxis Education for the Preventative Medicine Plus One Maternal Health Track

**DOI:** 10.1093/ofid/ofae631.1154

**Published:** 2025-01-29

**Authors:** Kristin A Carr, Alexander Thomas, Arshley Fleuristal, Jonathan Goorhuis

**Affiliations:** Loma Linda University Medical Center, Loma Linda, California; Loma Linda University Medical Center, Loma Linda, California; Loma Linda University Medical Center, Loma Linda, California; Loma Linda University Medical Center, Loma Linda, California

## Abstract

**Background:**

As HIV prevention methods continue to develop with new agents added for Pre-Exposure Prophylaxis (PrEP). Prep uptake has remained low among US women since its approval in 2012. One major barrier to increasing PrEP uptake remains lack of knowledge of PrEP among patients and providers and lack of access to PrEP care in regions with high HIV prevalence. This study aims to increase PrEP knowledge and medical competence among Maternal Health Track Preventive Medicine residents at a single academic center in Southern California.

Pre-Test and Post-Test Scores and % Improvement

**Figure:**
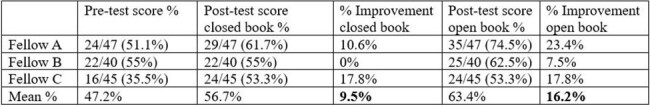

A pre-test was given at the start of each module. The same questions were made into a post-test after the curriculum was completed. Open and closed book portions were offered, and the % improvement was recorded.

**Methods:**

Three Preventative Medicine Fellows from Family Medicine and Obstetrics and Gynecology backgrounds enrolled in multidisciplinary curriculum involving women’s health. A series of twelve case-based lectures were completed from July 2023 through December 2023. Each four-hour session included a pre-test, lecture, and a problem-based learning module. Simultaneous, they provided direct care for women in a combined clinic for PrEP and HIV care weekly for 3-6 months. In March 2024, the pre-test questions were combined into 47 post-test questions.

**Results:**

Two of the three fellows had improvement in the closed book portion of the exam with a mean improvement of 9.5%. However, all of them had significant improvement for the open book exam with an average improvement of 16.2%. This suggests that although the memory retention of the material can wane with time, the ability to use resources to address clinically challenging questions has been strengthened. Of note, the questions were designed to simulate the American Academy of HIV Medicine (AAHIV) certification exam, which is an open-book test.

**Conclusion:**

The combination of lectures, self-assessments, case-based discussions, and clinical experience has enhanced the fellows’ knowledge of PrEP care for women based on the improvement of exam scores after the curriculum. This will enhance their ability to provide multidisciplinary preventative care for women in underserved areas.

**Disclosures:**

**Kristin A. Carr, MD**, Preventive Medicine Plus One Maternal Health Track: Grant/Research Support

